# Prognostic analysis of HALP -LDH composite model in newly diagnosed diffuse large B-cell lymphoma patients

**DOI:** 10.3389/fphys.2026.1877689

**Published:** 2026-10-30

**Authors:** Yugang Yan, Kewen Li, Qinhua Yu, Liannv Qiu

**Affiliations:** 1 Department of Clinical Laboratory, Quzhou Hospital of Traditional Chinese Medicine, Quzhou, Zhejiang, China; 2 Laboratory Medicine Center, Department of Clinical Laboratory, Zhejiang Provincial People's Hospital (Affiliated People's Hospital), Hangzhou Medical College, Hangzhou, Zhejiang, China; 3 School of Medical Technology and Information Engineering, Zhejiang Chinese Medical University, Hangzhou, Zhejiang, China

**Keywords:** diffuse large B cell lymphoma, HALP score, lactate dehydrogenase, prognosis, survival

## Abstract

**Background:**

The hemoglobin–albumin–lymphocyte–platelet (HALP) score is a prognostic marker in several types of malignant tumors. The prognostic value of HALP -Lactate dehydrogenase (LDH) composite model in diffuse large B-cell lymphoma (DLBCL) remains unknown.

**Objective:**

We aim to determine the prognostic value of the HALP +LDH composite model in DLBCL patients.

**Methods:**

We retrospectively analyzed their clinical data and laboratory parameters from 112 newly diagnosed DLBCL patients treated with R-CHOP regimens at Zhejiang Provincial People’s Hospital from January 2014 to October 2021. According to HALP score, these patients were divided into a high-HALP group (HALP score>24.92, n=65) and a low-HALP group (HALP score ≤ 24.92, n=47). We evaluated the significance of baseline HALP +LDH composite model as a predictor of response to treatment, overall survival (OS), progression-free survival (PFS) and clinicopathological characteristics. The risk score formula was generated based on the regression coefficients of model.

**Results:**

The median follow-up duration for the entire cohort was 36 months. DLBCL patients with a low HALP score (≤24.92) exhibit significantly adverse clinical and biological features, including advanced Ann Arbor stage (III–IV), elevated International Prognostic Index (IPI) score (3–5), LDH ≥250U/L, and ≥2 extranodal sites of involvement (all P < 0.05). A low HALP score (≤24.92) effectively identifies high-risk DLBCL patients and serves as an independent predictor of poor OS and PFS beyond other clinical characteristic, IPI. Additional independent predictors identified in the Cox regression analysis included advanced Ann Arbor stage III–IV, poor performance status (ECOG score ≥2), IPI score 3–5, LDH ≥250U/L and HALP (≤24.92). Furthermore, two combined models (HALP + LDH and HALP + IPI) significantly improves the assessment of patient prognosis (AUC = 0.717), superior to the individual predictive value of HALP score, LDH, or IPI alone.

**Conclusion:**

The HALP+LDH composite model enhances prognostic accuracy in DLBCL and complements conventional scoring systems.

## 1 Introduction

Diffuse large B-cell lymphoma (DLBCL), a highly aggressive hematological malignancy originating from mature B lymphocytes, is the most common type of non-Hodgkin lymphoma (NHL), accounting for approximately 30% of all cases worldwide (Swerdlow et al., 2016; Sukswai et al., 2020; Sehn and Salles, 2021). It predominantly affects middle-aged and elderly individuals, with a slight male predominance. DLBCL exhibits high tumor heterogeneity, encompassing significant differences at both the morphological and molecular levels, which leads to considerable individual variations in clinical manifestations, treatment responses, and prognoses (Zhang et al., 2023). Based on gene expression profiles, it can be further classified into germinal center B-cell-like (GCB), activated B-cell-like (ABC), and unclassified subtypes, each of which has differential response patterns to chemotherapy and targeted drugs (Reddy et al., 2017). Currently, first-line treatment with R-CHOP regimens (rituximab, cyclophosphamide, adriamycin, vincristine, and prednisone) has significantly prolonged progression-free survival (PFS) and overall survival (OS) in DLBCL patients (Zhang et al., 2023; Al-Sarayfi et al., 2024). Nevertheless, approximately 40% of DLBCL patients treated with R-CHOP still develop relapsed/refractory DLBCL. These poor outcomes may be associated with multiple biological and clinical factors, such as gene alterations, physical fitness, and immune function (Mondello and Mian, 2019; Wang and Li, 2020). Given the pronounced molecular heterogeneity and interpatient variability inherent to DLBCL, risk stratification remains challenging. Consequently, there is an urgent need to develop a robust, clinically applicable risk classification system that enables early identification of high-risk patients and facilitates personalized therapeutic decision-making to optimize survival outcomes.

In recent years, risk stratification for DLBCL has evolved substantially, with the International Prognostic Index (IPI) and the National Comprehensive Cancer Network–IPI (NCCN-IPI) remaining the most widely adopted clinical tools for prognostication and therapeutic decision-making (A predictive model for aggressive non-Hodgkin's lymphoma, 1993; Sehn et al., 2007). However, prognostic stratification systems of DLBCL, such as IPI, and NCCN-IPI, has demonstrated limited capacity in effectively identifying high-risk patients. These both conventional scoring systems rely primarily on clinical variables such as age, Ann Arbor stage, serum lactate dehydrogenase (LDH) level, number of extranodal involvement, and (in the case of NCCN-IPI) serum albumin level, and do not incorporate biologically relevant parameters such as nutritional status, systemic inflammatory response, or immune functional alterations, all of which have been increasingly recognized as independent determinants of prognosis (Zhou et al., 2014; Ruppert et al., 2020). This oversight may result in certain high-risk patients not being accurately identified, ultimately impacting their treatment and outcomes. Accumulating evidence further demonstrates that these host-derived parameters actively shape the tumor microenvironment, influence immunosurveillance efficacy, and modulate chemotherapy sensitivity, thereby serving as functionally relevant, non-redundant prognostic determinants (Rungwittayatiwat et al., 2021; Wang et al., 2024). Thus, identifying clinical available, cost-effective prognostic parameters are essential both for timely risk stratification and for guiding individualized treatment strategies in DLBCL.

Emerging evidence suggests that pre-treatment nutritional and systemic inflammatory markers (such as serum albumin) and derived composite scores (such as the prognostic nutritional index, PNI) exhibit significance prognostic value in various solid tumors, including hepatocellular carcinoma and penile cancer (Peng et al., 2025). Notably, the HALP score integrates hematologic, nutritional, and immunoinflammatory parameters, thereby comprehensively reflecting the overall nutritional status, systemic immune function, and bone marrow hematopoietic reserve capacity of patients. Previous studies have preliminarily verified the prognostic value of the HALP score in various solid tumors and hematological malignancies (Xu et al., 2020; Dagmura et al., 2022; Dong et al., 2024). LDH, as a key rate-limiting enzyme catalyzing the interconversion between pyruvate and lactate, is a core regulatory molecule mediating tumor aerobic glycolysis (Warburg effect) (de la Cruz-López et al., 2019). Serum LDH levels correlate strongly with tumor burden, proliferation, and metabolic activity. Clinical studies have confirmed that high LDH levels usually indicate increased resistance of tumor cells to chemotherapy, targeted therapy, and immunotherapy (Hirami et al., 2023). In DLBCL, elevated LDH is a core component of the NCCN-IPI scoring system, indicating that patients belong to the high-risk prognostic group (Zhou et al., 2014; Liu et al., 2019). High baseline LDH is a validated independent prognostic biomarker for poor survival in some tumor (Dong et al., 2017; Cui et al., 2020; Zhang et al., 2025). In mantle cell lymphoma (MCL), combining LDH with standard clinical factors improves recurrence risk prediction (Hoster et al., 2008). In small cell lung cancer (SCLC), LDH levels reflect tumor stage, metastatic extent, and prognosis. However, the prognostic utility of HALP combined application with LDH and its clinical relevance in DLBCL still require in-depth exploration.

To address these limitations, we developed the HALP+LDH model grounded exclusively on routinely available peripheral blood biomarkers to enhance the precision of risk prediction in DLBCL.

## 2 Materials and methods

### 2.1 Research objects

This study was a single-center retrospective cohort study that included 112 patients with newly diagnosed DLBCL who were treated at Zhejiang Provincial People’s Hospital from January 2014 to October 2021. The following strict inclusion criteria were adhered to: (1) All patients met the diagnostic criteria of the World Health Organization (WHO) Classification of Tumors of Hematopoietic and Lymphoid Tissues (Alaggio et al., 2022); (2) The patient was newly diagnosed and had not received any treatment for DLBCL to ensure the accuracy of HALP score which would not be affected by previous treatment; and (3) Patients had the required laboratory indicator data labor.

Patients with any of the following conditions were excluded: (1) concurrent malignancy or autoimmune disease; (2) active infection, including acute systemic infection, chronic hepatitis B virus (HBV) infection, or other clinically significant infectious conditions; (3) coagulopathy or moderate-to-severe impairment of cardiac, hepatic, or renal function (e.g., estimated glomerular filtration rate <60 mL/min/1.73 m², Child–Pugh class B or C, or New York Heart Association class ≥III); (4) pregnancy or lactation; and (5) severe neuropsychiatric or cognitive disorders.

### 2.2 Treatment regimens

All the patients included in this study received the immunotherapy chemotherapy regimen R-CHOP based on rituximab. The R-CHOP regimen consists of rituximab (375 mg/m² on day 0), doxorubicin liposome (50 mg/m² on day 1), cyclophosphamide (750 mg/m² on day 1), vincristine (1.4 mg/m² on day 1), and prednisone (100 mg from day 1 to day 5). This regimen is carried out in 21-day cycles for a total of 6 cycles.

### 2.3 Data collection

Diagnosis, age, gender, Ann Arbor stage, extranodal involvement, ECOG performance status, Hans immunohistochemical classification, laboratory parameters: hemoglobin, lymphocyte, platelet, albumin, serum albumin and LDH level were collected from electronic medical records at the initial diagnosis and before treatment. The HALP score = [hemoglobin (g/L) × albumin (g/L) × absolute lymphocyte count (×10^9^/L)]/platelet count (×10^9^/L).

The outcomes analyzed were response to treatment, PFS, and OS. Treatment responses were assessed according to the Lugano classification (Cheson et al., 2014). The objective response rate (ORR) was calculated as the proportion of patients achieving either partial response (PR) or complete remission (CR) at the end of treatment: ORR = (CR + PR)/total evaluable patients ×100%. PFS was defined as the time from initial diagnosis to the first occurrence of disease progression (relapsed or refractory disease) or death from any cause, and OS was defined as the time from initial diagnosis to death or last follow-up.

### 2.4 Construction of the composite risk model

To develop the composite risk model, a Cox regression model was fitted using HALP score (≤24.92 vs >24.92), LDH level (≤250 vs >250 U/L) and IPI (3–5 vs 0–2) as covariates to generate a combined predictive probability for each patient. The optimal cutoff value for dichotomizing patients into low-risk and high-risk groups was determined using the maximally selected log-rank statistic (minimum P value approach). Patients with a predictive probability above the cutoff were classified as high-risk, and those below or equal to the cutoff as low-risk. The prognostic value of th is risk stratification was further validated in a stratified Cox regression model adjusted for ECOG performance status.

With OS and PFS set as the endpoints, multivariate Cox proportional hazards regression prognostic models were constructed. Separate combined models (HALP + LDH and HALP + IPI) and a single-variable IPI control model were constructed. Separate combined models (HALP + LDH and HALP + IPI) and a single-variable IPI control model were constructed. The risk score formula was generated based on the regression coefficients of each model. Bootstrap resampling was performed to correct the C-index for assessing the discrimination ability of each prognostic model.

### 2.5 Statistical analysis

All statistical analyses were performed using the GraphPad Prism 10.6 and SPSS Statistics version 24.0. Categorical variables are presented as percentages [n (%)], and intergroup comparisons were conducted using the chi-square (χ²) test where appropriate. Kaplan Meier method was used to draw the survival curve. Univariable and multivariable Cox proportional hazards regression models were employed to identify independent prognostic factors associated with OS and PFS. A two-sided P < 0.05 was considered statistically significant.

## 3 Results

### 3.1 The relationship between HALP score and clinical pathological characteristics

A total of 176 cases were initially screened in this study. After excluding duplicate records and strictly adhering to the inclusion criteria, 112 patients with newly diagnosed DLBCL were finally included for analysis (**Figure 1**). The baseline characteristics of the patients are as follows (**Table 1**): 71 patients (63.39%) were older than 60 years; 69 patients (61.61%) were male and 43 patients (38.39%) were female; 68 patients (60.71%) were at Ann Arbor stage III/IV; 20 patients (17.86%) exhibited poor performance status (ECOG ≥ 2). According to the Han classification, 71 patients (63.39%) were non-GCB and 41 (36.61%) were GCB.

**Figure 1:**
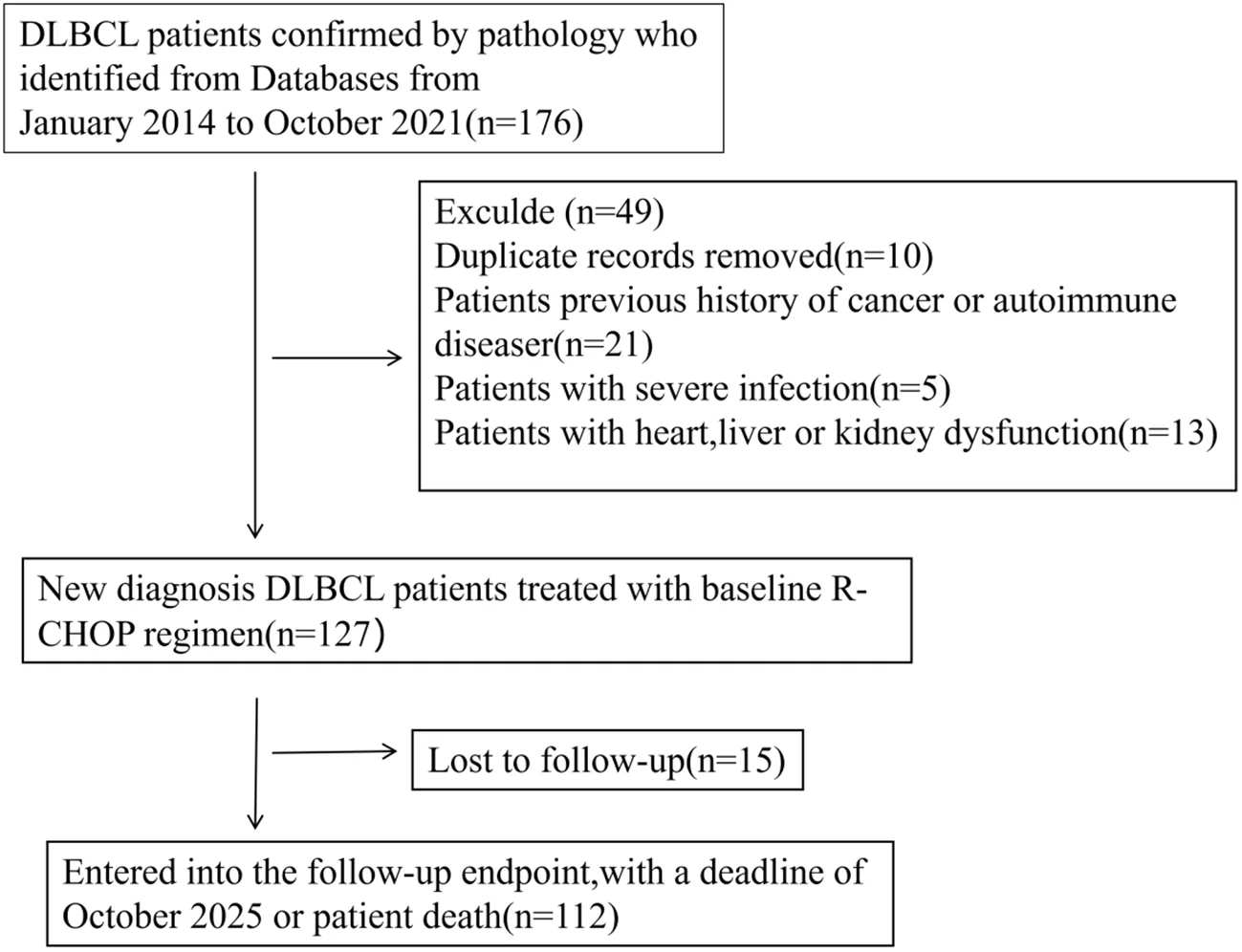
Flow chart of patient selection.

**Table 1:** The relationship between HALP score and clinical pathological characteristics [n, (%)].

Variable	Total (n = 112),	HALP			χ2	*P*
		>24.92, (n=65)	≤24.92, (n=47)			
Age (years)					2.297	0.095
> 60	71 (63.39)	37 (56.92)		34 (72.34)		
≤60	41 (36.61)	28 (40.38)		13 (27.66)		
Gender					1.437	0.2307
Male	69 (61.61)	37 (56.92)		32 (68.09)		
Female	43 (38.39)	28 (43.08)		15 (31.91)		
Ann Arbor staging					8.564	0.0034
I–II	44 (39.29)	33 (50.77)		11 (23.40)		
III–IV	68 (60.71)	32 (49.23)		36 (76.60)		
ECOG score					0.4849	0.619
0–1	92 (82.14)	52 (80.00)		40 (85.11)		
≥2	20 (17.86)	13 (20.00)		7 (14.89)		
LDH level (U/L)					6.792	0.0092
≤250	66 (58.93)	45 (69.23)		21 (44.68)		
>250	46 (41.07)	20 (30.77)		26 (55.32)		
Number of extranodal involvement					5.389	0.0271
0-1	84 (75.00)	54 (83.08)		30 (63.83)		
IPI score					8.973	0.0027
0–2	66 (58.93)	46 (70.77)		20 (42.55)		
3–5	46 (41.07)	19 (29.23)		27 (57.45)		
Hans classification					1.234	0.2667
GCB	41 (36.61)	21 (32.31)		20 (31.91)		
Non-GCB	71 (63.39)	44 (67.69)		27 (68.09)		
Treatment outcome					9.804	0.0021
Response CR/PR	88 (78.57)	57 (87.69)		31 (65.96)		
No response SD/PD	24 (21.43)	8 (12.31)		16 (34.04)		

ECOG, Eastern Cooperative Oncology Group; IPI, International Prognostic Index; GCB, germinal center B-cell-like; LDH, lactate dehydrogenase; HALP, hemoglobin–albumin–lymphocyte–platelet; CR, complete remission; PR, partial response; SD, stable disease; PD, progressive disease.

The median HALP score of the 112 patients was 27.80 (interquartile range [IQR]: 14.66 - 43.76). To select the optimal prognostic cut-off value, we performed receiver operating characteristic (ROC) analysis and maximized the log-rank test statistic using GraphPad Prism. Both approaches yielded consistent results: the optimal cut-off value for predicting OS or PFS was 24.92 or 27.80. Given superior discriminatory power for OS and consistent with the conclusions of previous studies in hematologic malignancies, a HALP cut-off of 24.92 was selected for primary analysis. Accordingly, patients were classified into a high-HALP group (HALP > 24.92; n = 65) and a low-HALP group (HALP ≤ 24.92; n = 47).

Comparative analysis revealed statistically significant differences between the two HALP groups in Ann Arbor stage, LDH level, number of extranodal involvement and IPI score. No significant differences were observed in Age, Gender, ECOG performance status, or Hans classification, as shown in **Table 1**. Patients with advanced Ann Arbor Stage (III-IV), high IPI score, high number of extranodal involvement, and high LDH level had lower HALP score, suggesting that HALP score is negatively correlated with tumor invasiveness.

The overall response rate (ORR) was significantly lower in the low-HALP group (65.96%) than in the high-HALP group (87.69%, **Table 1**). The no response rate (SD+PD) was significantly higher in the low-HALP group (34.04%) than in the high-HALP group (12.31%), suggesting that HALP score is negatively associated with treatment response.

### 3.2 Effect of HALP score on OS and PFS of patients with DLBCL

The median follow-up duration for the 112 enrolled patients in this study was 34.5 months (range: 2–125 months), with an average follow-up of 50 months. During follow-up, a total of 50 patients died. Survival analyses demonstrated that patients in the high-HALP group had significantly better PFS (**Figure 2A**) and OS (**Figure 2B**) than those in the low-HALP group (chi-square test: PFS, χ² = 7.171, *P* = 0.007; OS, χ² = 10.200, *P* = 0.001).

**Figure 2:**
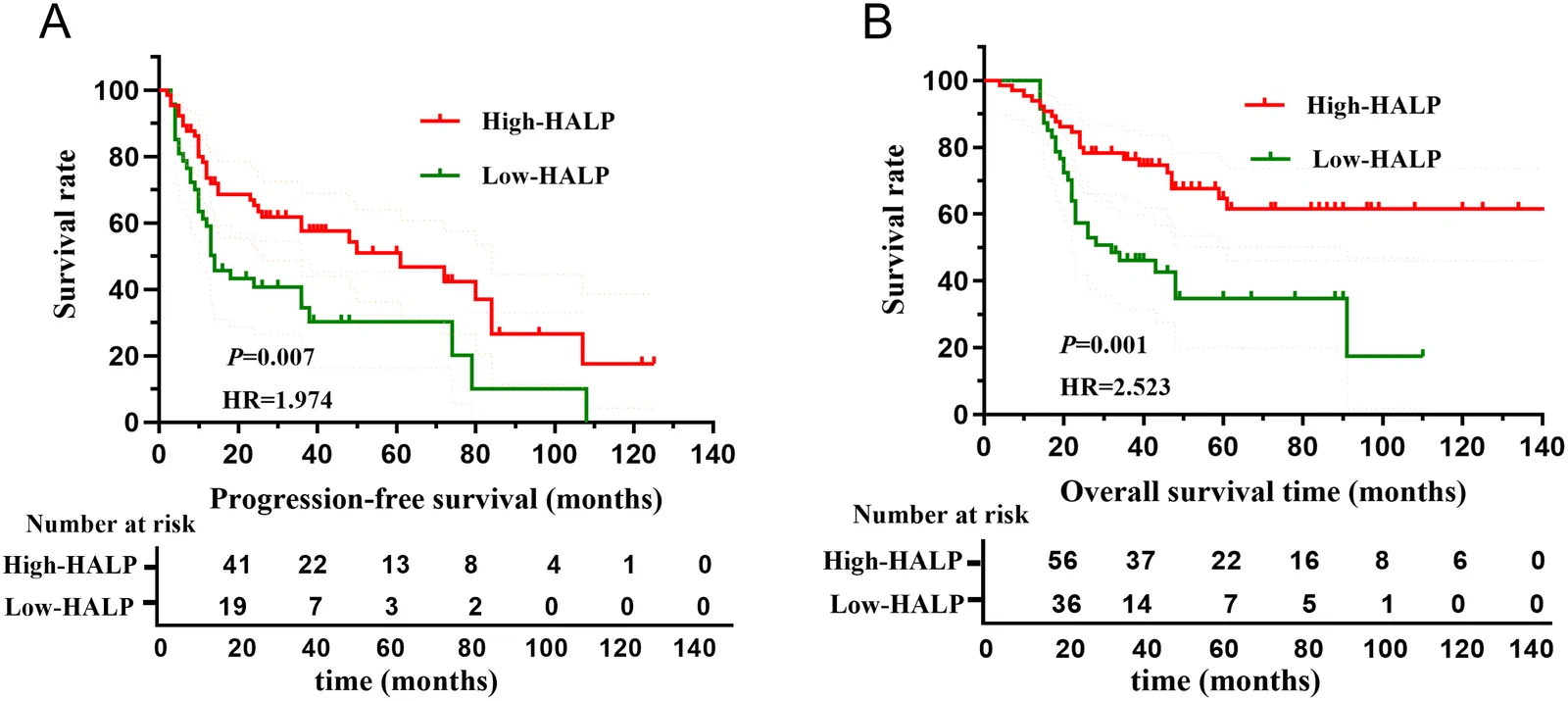
Comparison of PFS and OS between low- and high- HALP group. **(A)** PFS; **(B)** OS.

For the high-HALP group, the 20-, 40-, 60-, 80-, 100-, and 120-month PFS rates were 66.86% (95% CI: 53.81%-79.92%), 57.50% (95% CI: 43.91%-71.09%), 50.91% (95% CI: 36.15%-65.67%), 37.12% (95% CI 20.76%-53.49%), 26.52% (95% CI 11.37%-41.66%), 17.68% (95% CI 4.30%-31.06%), with 41, 22, 13, 8, 4 and 1 patients at risk at each time point, respectively. In contrast, the corresponding PFS rates for the low-HALP group were 43.32% (95% CI: 28.76%-57.88%), 30.18% (95% CI: 16.29%-44.08%), 30.18% (95% CI: 16.29%-44.08%), 10.06% (95% CI: 0-33.06%), and 0% at both 100 and 120 months (not estimable), with 19, 7, 3, 2 and 0 patients at risk, respectively. The median PFS was 61.0 months in the high-HALP group versus 14.0 months in the low-HALP group (HR = 1.974, 95% CI: 1.196-3.170, P = 0.007) (**Figure 2A**).

The 20-, 40-, 60-, 80-, 100- and 120-month OS rates for the high-HALP group were 86.13% (95% CI: 79.72%-97.22%), 74.61% (95% CI: 65.58%-83.65%), 64.68% (95% CI: 50.00%-79.35%), 61.44% (95% CI: 46.08%-76.81%), 61.44% (95% CI: 46.08%-76.81%), and 61.44% (95% CI: 46.08%-76.81%), with 56, 37, 22, 16, 8 and 6 patients at risk, respectively. The corresponding 20-, 40-, 60-, 80- and 100-month OS rates for the low-HALP group were 72.34% (95% CI: 61.78%-87.50%), 46.12% (95% CI: 32.60%-59.64%), 34.83% (95% CI: 19.52%-50.14%), 34.83% (95% CI: 19.52%-50.14%), and 17.42% (95% CI: 0-46.78%), with 36, 14, 7, 5 and 1 patients at risk, respectively. No patients remained at risk in the low-HALP group at 120 months. The median OS was not reached in the high-HALP group, compared with 32.0 months in the low-HALP group (HR = 2.523, 95% CI: 1.430–4.451, P = 0.001) (**Figure 2B**).

In conclusion, the HALP score is a reliable and clinically significant prognostic biomarker in DLBCL patients, with higher HALP scores consistently associated with better short-term and long-term survival outcomes.

### 3.3 Univariate and multivariate analysis of prognostic factors of DLBCL patients

Univariate Cox regression analysis revealed that HALP Ann Arbor stage, ECOG physical status score, LDH levels, Number of extranodal involvement, and IPI score were significant predictors of OS in DLBCL patients. Multivariable Cox regression analysis identified LDH level and HALP score as independent prognostic factors for OS (**Supplementary Table 1**). To minimize potential bias caused by dichotomization, we further conducted Cox regression analysis using HALP and LDH as continuous variables for OS; these continuous-variable-based results are presented in **Table 2**.

**Table 2:** Prognostic factor analysis for OS by Cox regression: HALP and LDH treated as continuous variable.

Indicator	Univariate analysis			Multivariate analysis					
				Without IPI score			Plus IPI score		
	HR	*P*	95% CI	HR	*P*	95% CI	HR	*P*	95% CI
HALP score	0.983	0.024*	0.968~0.998	0.983	0.037*	0.968~0.999	0.987	0.098	0.972~1.002
Age (> 60 years *vs*. ≤60 years)	1.350	0.311	0.750~2.427	–	–	–	–	–	–
Gender (male *vs*. female)	1.391	0.247	0.796~2.433	–	–	–	–	–	–
Ann Arbor stage (stage III–IV *vs*. stage I–II)	1.930	0.037*	1.039~3.584	0.833	0.668	0.361~1.923	–	–	–
ECOG physical status score (≥2 points *vs*. 0–1 point)	2.212	0.010*	1.206 ~4.049	–	–	–	–	–	–
LDH levels	1.001	P<0.001*	1.000~1.001	1.001	0.007*	1.000~1.001	–	–	–
Number of extranodal involvement (≥2 *vs*. 0–1)	1.965	0.027*	1.080~3.571	1.737	0.129	0.851~3.545	–	–	–
IPI score (3–5 points *vs*. 0–2 points)	3.268	<0.001*	1.842~5.814	–	–	–	2.489	0.010*	1.241~4.994
Hans classification (GCB subtype *vs*. non-GCB subtype)	0.877	0.654	0.495~1.555	–	–	–	–	–	–

OS, overall survival; HR, risk ratio; CI, confidence interval; HALP, hemoglobin-albumin-lymphocyte-platelet; ECOG, Eastern Cooperative Oncology Group; LDH, lactate dehydrogenase; IPI, International Prognostic Index;GCB, germinal center B-cell-like; *, P<0.05.

For PFS, univariate Cox regression analysis identified HALP, LDH levels and IPI score as significant predictors. Multivariable Cox regression analysis also confirmed LDH level and HALP as independent prognostic factor for PFS (**Supplementary Table 2**). We further evaluated HALP and LDH as a continuous variable in multivariable Cox regression to circumvent bias arising from categorical grouping and demonstrated LDH level as an independent prognostic factor for PFS, whereas HALP failed to show independent predictive significance (**Table 3**).

**Table 3:** Prognostic factor analysis for PFS by Cox regression: HALP and LDH treated as continuous variable.

Indicator	Univariate analysis			Multivariate analysis			Multivariate analysis		
				Without IPI score			Plus IPI score		
	HR	*P*	95% CI	HR	*P*	95% CI	HR	*P*	95% CI
HALP score	0.992	0.183	0.981~1.004	0.993	0.259	0.982~1.005	0.994	0.346*	0.983~1.006
Age (> 60 years *vs*. ≤60 years)	1.599	0.077	0.951~2.689	–	–	–	–	–	–
Gender (male *vs*. female)	0.808	0.392	0.497~1.315	–	–	–	–	–	–
Ann Arbor stage (stage III–IV *vs*. stage I–II)	1.266	0.356	0.767~2.090	–	–	–	–	–	–
ECOG physical status score (≥2 points *vs*. 0–1 point)	1.585	0.110	0.901~2.790	–	–	–	–	–	–
LDH levels	1.001	0.004*	1.000~1.001	1.001	0.006*	1.000~1.001			
Number of extranodal involvement (≥2 *vs*. 0–1)	1.645	0.069	0.962~2.812	–	–	–	–	–	–
Gender (male *vs*. female)	0.808	0.392	0.497~1.315	–	–	–	–	–	–
IPI score (3–5 points *vs*. 0–2 points)	2.083	0.003*	1.285~3.389	–	–	–	2.015	0.005*	1.234~3.290
Hans classification (GCB subtype *vs*. non-GCB subtype)	1.019	0.942	0.615~1.687	–	–	–	–	–	–

PFS, progression-free survival; HR, risk ratio; CI, confidence interval; HALP, hemoglobin-albumin-lymphocyte-platelet; ECOG, Eastern Cooperative Oncology Group; LDH, lactate dehydrogenase hemoglobin; IPI, International Prognostic Index; GCB, germinal center B-cell-like; *, P<0.05.

### 3.4 ROC curve analysis of HALP + LDH model

The Kaplan-Meier analysis demonstrated that a low HALP score and high LDH level were associated with poorer OS and PFS in DLBCL patients. To quantitatively assess prognostic discrimination, ROC curve analysis was performed for five models: HALP score alone, LDH level alone, the IPI score, and two composite models (HALP+LDH and HALP+IPI) (**Table 4**, **Figure 3**). All models demonstrated statistically significant discriminatory utility (HALP, *P* = 0.003; LDH, *P* = 0.0006; IPI, *P* = 0.0007; HALP+LDH model, *P* < 0.0001; HALP+IPI model, *P* < 0.001). Among them, the HALP+LDH and HALP+IPI combined model both achieved an AUC of 0.717, significantly higher than that of HALP alone (0.663), LDH alone (0.690), and the established IPI score (0.687).

**Table 4:** Prediction ability of HALP, LDH, IPI for prognosis of DLBCL patients.

Variables	AUC	*P*	95% CI	Sensitivity	Specificity	DeLong-p (HALP+LDHvs/)
HALP	0.663	0.0030	0.557-0.770	58%	71%	<0.01
LDH	0.690	0.0006	0.590-0.790	72%	63%	0.437
IPI score	0.687	0.0007	0.587-0.786	60%	74%	0.509
HALP +LDH	0.717	<0.0001	0.633-0.823	75%	68%	Reference
HALP+IPI	0.717	<0.001	0.620-0.814	76%	55%	0.997

AUC, area under the ROC curve; CI, confidence interval; HALP, hemoglobin–albumin–lymphocyte–platelet; LDH, lactate dehydrogenase; IPI, International Prognostic Index.

**Figure 3:**
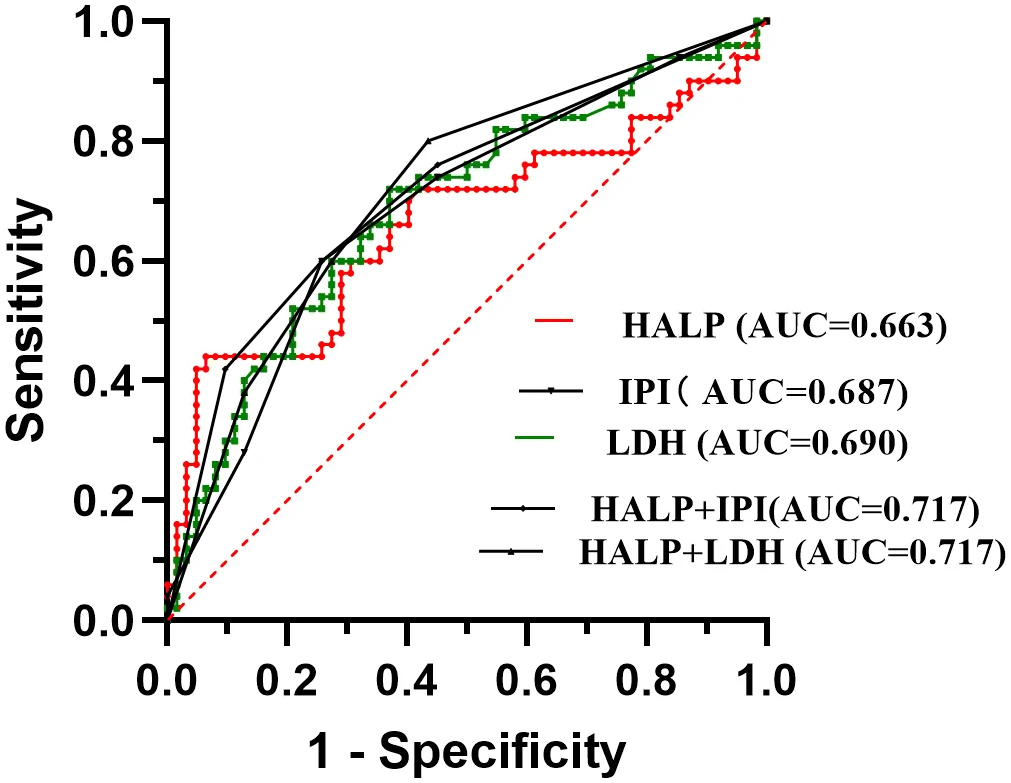
ROC curve analysis of the predictive value of HALP score, IPI, LDH, LDH+IPI and HALP+LDH for prognosis of DLBCL patients.

A weighted prognostic risk score was derived from multivariate Cox proportional hazards regression, incorporating HALP and LDH as independent predictors. Using maximally selected rank statistics, an optimal cut-off value of 0.24065 was identified to dichotomize patients into high- and low-risk groups. Notably, patients in the high-risk group exhibited significantly inferior median OS than those in the low-risk group (35.0 months, 95% CI: 16.272–53.728 vs. not reached; log-rank χ² = 16.131, *P* < 0.001). In a stratified Cox regression model adjusted for ECOG performance status, HALP+LDH risk stratification remained an independent prognostic factor for OS (HR = 3.268, 95% CI: 1.591–6.716, *P* = 0.001), whereas Ann Arbor stage and number of extranodal sites did not retain independent prognostic significance (both *P* > 0.05) (**Supplementary Table 4**).

Regarding the clinical utility, LDH exhibited the highest sensitivity (72%) but relatively moderate specificity (63%); the IPI score achieved the highest specificity (74%), rendering it particularly suitable for reliably identifying low-risk patients; HALP alone showed the lowest sensitivity (58%) yet maintained favorable specificity (71%), supporting its role as a complementary prognostic adjunct. Critically, the HALP+LDH combination preserved high specificity (68%) while improving sensitivity to 75%, thereby enhancing both case detection and risk stratification accuracy. HALP reflect the nutritional immune state whereas LDH serves as a surrogate marker of tumor burden. Their integration realizes the comprehensive evaluation of the prognosis of the DLBCL patient from two dimensions of host state and tumor characteristics. This balanced diagnostic profile positions the combined model as an economical, widely accessible, and clinically meaningful tool for refined risk stratification in DLBCL.

To avoid optimistic bias introduced by dichotomization based on cutoff values, we further developed comparative prognostic models with HALP and LDH treated as continuous variables, including the HALP+LDH model, IPI-alone model, and the combined HALP with IPI. The corresponding Cox regression coefficients, hazard ratios and bootstrap-optimism-corrected C-index for OS and PFS are shown in **Table 5**. For OS, the bootstrap-corrected C-index was 0.663 for HALP+LDH model, 0.641 for IPI-alone model, and 0.679 for HALP+IPI combined model. For PFS, the bootstrap-corrected C-index values were 0.613, 0.588 and 0.605, respectively. In addition, time-dependent AUC and Brier scores at clinically relevant time points (1-year and 3-year) for these models are provided in **Supplementary Table 3**. Adding HALP to IPI yielded a modest improvement in discriminative performance for OS, while limited improvement was observed for PFS.

**Table 5:** Multivariate Cox proportional hazards regression analysis and bootstrap-corrected C-index of different prognostic models for OS and PFS.

Endpoint	Model	Variable	β coefficient	HR	95%CI	Bootstrap-optimism corrected *C*-index
OS	HALP+LDH	HALP LDH	-0.4651 0.1337	0.628 1.143	0.408-0.967 1.06-1.250	0.663
	IPI	IPI	1.1969	3.310	1.866-5.872	0.641
	HALP+IPI	HALP IPI	-0.3730 1.1145	0.689 3.048	0.451-1.051 1.702-5.460	0.679
PFS	HALP+LDH	HALP LDH	-0.1958 0.1281	0.822 1.137	0.589-1.148 1.040-1.242	0.613
	IPI	IPI	0.7492	2.115	1.303-3.435	0.588
	HALP+IPI	HALP IPI	-0.1668 0.7144	0.846 2.043	0.605-1.184 1.252-3.335	0.605

Multivariate Cox regression confirmed HALP as an independent protective factor for OS (HR = 0.628; 95% CI: 0.408–0.967), whereas both LDH and the IPI score were identified as independent risk factors for OS and PFS. However, in all multivariate models evaluating PFS, included unity (i.e., HR crossing 1), indicating insufficient evidence for HALP to serve as an independent predictor of disease progression. Accordingly, the final OS-specific risk score for the HALP+LDH model is defined as: *Risk score = −0.4651 × HALP + 0.1337 × LDH*.

Internal validation using bootstrap-corrected C-indices further supported the model’s discriminative performance. For OS prediction, the HALP+IPI model yielded a C-index of 0.679—modestly superior to the IPI-alone model (C-index = 0.641). For PFS prediction, the HALP+LDH model attained the highest C-index (0.613). Importantly, integrating HALP into the IPI framework enhanced long-term OS discrimination, underscoring its potential as a biologically plausible, cost-effective adjunct biomarker in DLBCL prognostication.

## 4 Discussion

DLBCL is a highly heterogeneous hematological malignancy, characterized by rapid disease progression and significant systemic inflammatory responses, and frequent concomitant nutritional and metabolic disorders. While approximately 60% of patients achieve sustained remission with standard first-line immunotherapy such as R-CHOP, a considerable proportion of patients still develop drug resistance or relapse (Zhang et al., 2022).Traditional clinical risk stratification systems like IPI, while widely used in clinical practice, have inherent limitations in accurately identifying high-risk patients and predicting prognosis due to their reliance on clinical parameters including age, stage, LDH level, performance status and extranodal involvement sites,. Systemic inflammatory response and nutritional metabolic disorders have been confirmed as key host-related factors influencing tumor initiation, progression, treatment tolerance, and ultimate survival outcomes, and have been identified as independent adverse prognostic factors in DLBCL, apart from traditional clinical parameters. Epidemiological data show that 40% to 80% of patients with solid tumors and hematological malignancies exhibit varying degrees of nutritional disorders, which correlates significantly with decreased treatment tolerance, increased complication rates, deteriorated quality of life, and multiple organ dysfunction (Wang et al., 2023).

Hemoglobin not only characterizes the tissue oxygenation capacity but also indirectly reflects the chronic inflammatory burden by regulating erythropoiesis and oxidative stress levels; Albumin is both a nutritional indicator and also exerts endogenous anti-inflammatory effects by specifically binding and neutralizing pro-inflammatory cytokines such as IL-6 and TNF-α (Zhang et al., 2023). For patients after radical gastrectomy, the dynamic decline in hemoglobin and albumin levels correlate negatively with the severity of early postoperative complications; both have been confirmed as independent predictors of OS, and their combined application yields significantly better prognostic discrimination than either single indicator (Ai et al., 2019).

Lymphopenia reflects impaired cellular immune function and is mechanistically linked to reduced tumor immune surveillance, enhanced angiogenesis, and increased metastatic potential. In DLBCL, decreased absolute lymphocyte count (ALC) at initial diagnosis is closely related to Ann Arbor stage III–IV, high-risk IPI scores (Swerdlow et al., 2016; Reddy et al., 2017; Zhang et al., 2023), reduced chemotherapy response rates, and inferior OS and PFS, making it an important host marker for evaluating disease aggressiveness and primary drug resistance (Shi et al., 2024).

Platelets not only participate in the homeostasis process but also release factors such as vascular endothelial growth factor (VEGF) and platelet-derived growth factor (PDGF) to promote epithelial-mesenchymal transition (EMT) and the formation of immunosuppressive tumor microenvironment labor. Multiple cohort studies have confirmed that thrombocytopenia is an independent risk factor for shortened OS in patients with non-small cell lung cancer (NSCLC), suggesting that it can serve as a sensitive indicator of systemic host deterioration and adverse prognosis (Sandfeld-Paulsen et al., 2023).

Based on the synergistic pathophysiological basis of the above indicators in the occurrence and development of DLBCL, tumor microenvironment regulation and treatment response, this study innovatively constructed the HALP composite index and integrated it with LDH into the prognostic risk model framework to achieve systematic quantitative assessment of the bidirectional interaction between host and tumor. Based on the theoretical framework of “host-tumor bidirectional interaction”, this study innovatively constructed and validated a composite prognostic model that integrates the host-oriented biomarker HALP score.

The HALP score integrates four routine peripheral blood parameters: hemoglobin, serum albumin, absolute lymphocyte count, and platelet count, to form a composite biomarker reflective of systemic nutritional status, immune function, and inflammatory burden. Cross-tumor validation studies have confirmed its broad prognostic value: in bladder cancer, a cutoff value of 28.67 effectively distinguishes high-risk and low-risk groups in terms of OS (Yoldas et al., 2024); in renal cell carcinoma, the median OS of the low-HALP group is only 17.7 months, compared with while 89.7 months in the high-HALP group (Peng et al., 2018); in prostate cancer, a preoperative HALP score < 32.4 can independently predict an increased risk of biochemical recurrence (Kaya et al., 2021).

Here we recruited 112 newly diagnosed DLBCL patients to systematically evaluate the clinical correlation and prognostic efficacy of the HALP. Consistent with previous research (Cetintepe et al., 2024), a low HALP score (≤24.92) correlated significantly with high-risk clinical features of DLBCL, including Ann Arbor stage III-IV, IPI score 3-5, and >2 extranodal lesions, as well as inferior OS and PFS. Multivariate Cox regression confirmed HALP as an independent protective factor for OS but not for PFS—a dissociation attributable to fundamental differences between these endpoints. ROC analysis showed moderate to good discriminatory capacity for 3-year OS prediction: AUCs were 0.663 for HALP, 0.687 for IPI, and 0.690 for LDH. More importantly, stratified survival analysis demonstrated significantly inferior median OS and PFS in the low -HALP group compared with in the high-HALP group, confirming that HALP provides additional prognostic information beyond the IPI and highlighting its value as a complementary risk marker.

OS is strongly influenced by host-related determinants, including systemic inflammatory homeostasis, nutritional adequacy, and adaptive immune competence, that collectively modulate treatment tolerance, recovery potential, and late mortality. In contrast, PFS is predominantly governed by tumor-intrinsic proliferation kinetics and microenvironmental interactions less directly captured by HALP. Thus, HALP’s lack of independent association with PFS does not indicate methodological weakness but rather reflects its biologically appropriate domain of prognostic relevance: long-term survival outcomes.

This study establishes a composite prognostic model integrating HALP and LDH to advance risk assessment in DLBCL. HALP serves as a validated integrative marker of systemic inflammation, nutritional status, and host immune reserve, whereas LDH reflects tumor proliferative activity and metabolic burden. By jointly capturing host physiological resilience and tumor biological aggressiveness, the HALP+LDH model provides a mechanistically coherent, bidimensional prognostic framework, distinct from unidimensional clinical indices such as the IPI. Consistent with this biological rationale, the HALP+LDH model demonstrated statistically superior predictive accuracy for 3-year OS compared with HALP alone, LDH alone, and the conventional IPI.

This model integrates HALP and LDH via Cox hazards regression to generate a combined prediction risk probability. Using maximally selected rank statistics, an optimal cut-off of 0.24065 dichotomized patients into high- and low-risk groups. At this threshold, the model achieved an AUC of 0.717 for 3-year OS prediction—significantly exceeding those of individual markers and the conventional IPI. By integrating the HALP index and LDH, this model achieved complementary synergy in the dual-dimensional assessment of “host-oriented” and “tumor-oriented” factors, overcome the inherent limitations of single-parameter clinical models and provide an evidence-based foundation for refined risk stratification and individualized therapeutic decision-making in DLBCL.

The core advantage of the HALP score lies in its excellent clinical accessibility and health-economic efficiency: all constituent parameters are routine hematology and basic biochemical test items, no special instruments, additional costs or professional image interpretation capabilities are required; the detection process is highly standardized, the result has good repeatability, and is minimally affected by sampling time, operator differences and inter-laboratory variation; it is suitable for large-scale population screening and promotion in primary medical institutions, especially for areas with limited access to imaging technology and molecular detection resources. It is worth noting that reduced HALP essentially reflects the systemic imbalance of the “nutrition-immunity-inflammation axis”, and this pathophysiological process has been confirmed by a large number of basic and clinical studies to be involved in the occurrence and development of DLBCL, the shaping of immunosuppressive microenvironment, the formation of treatment resistance and poor prognosis (Peng et al., 2018; Yoldas et al., 2024). Although the AUC of HALP alone is modest (0.663), its threefold clinical advantages—accessibility, standardization, and biological plausibility—confer irreplaceable practical utility in routine oncology practice. LDH is a long-term clinically validated and mature tumor burden marker, and this study reconfirmed its good prognostic prediction efficacy (AUC = 0.690), and further demonstrates that the combination of HALP score with LDH significantly improves risk discrimination following first-line immunochemotherapy. We also found that combining HALP score with IPI can further improve the accuracy of prognosis judgment for DLBCL patients after first-line immunochemotherapy.

In comparison, although PET-based metabolic tumor volume (MTV) and deep learning-driven radiomic features (Jiang et al., 2022; Eertink et al., 2023; Jiang et al., 2023)show high accuracy in the prognostic assessment of DLBCL, their application is limited by equipment accessibility, examination costs and professional interpretation thresholds. Similarly, dynamic monitoring of circulating tumor DNA (ctDNA) has been validated as a sensitive predictor of treatment response and minimal residual disease (Li et al., 2022), however, its widespread adoption is hindered by high assay costs, lack of analytical standardization across laboratories, and insufficient validation in routine clinical workflows. Therefore, the HALP+LDH model is not proposed as a substitute for these advanced modalities, but rather as a pragmatic, complementary tool—designed to deliver rapid, cost-effective, and universally accessible risk screening and longitudinal monitoring, particularly within resource-stratified healthcare systems.

This study has several limitations. First, the single-center retrospective design and sample size (n=112) may introduce selection bias, and the external validity of its conclusion needs to be verified through multi-center, prospective cohort. Second, advanced molecular markers such as MYC/BCL2 double expression, TP53 gene mutation, and dynamic ctDNA clearance during treatment (Chen et al., 2023; Monick and Rosenthal, 2024) have not yet been integrated. Integrating such biologically refined markers is expected to further enhance discriminatory precision and biological interpretability of risk stratification (Wright et al., 2020).

In conclusion, this study confirms that the HALP score has independent prognostic value in DLBCL, and its combination with LDH can significantly improve the accuracy of risk stratification after first-line immunochemotherapy. The HALP+LDH composite model especially provide an economical, economically sustainable, and effective prognostic assessment tool for DLBCL patients in resource-limited settings. Future larger-scale multicenter studies are needed to further validate its clinical value and explore its integrated application with other advanced technologies to promote the development of individualized treatment for DLBCL.

## Data Availability

The original contributions presented in the study are included in the article/**Supplementary Material**. Further inquiries can be directed to the corresponding author/s.
