# Supplementary material for: Prognostic analysis of HALP -LDH composite model in newly diagnosed diffuse large B-cell lymphoma patients
Source: Front Physiol. 2026 Oct 30;17:1877689. doi: 10.3389/fphys.2026.1877689 (PMC13759408; doi:10.3389/fphys.2026.1877689)
Supplement: Supplementary file 1 [file Table1.docx]

Supplementary Material

**Supplementary Table 1.** Analysis of prognostic factors for OS in DLBCL patients

| **Indicator** | **Univariate analysis** | | | **Multivariate analysis** | | | | | |
| --- | --- | --- | --- | --- | --- | --- | --- | --- | --- |
|  |  | | | **without IPI score** | | | **plus IPI score** | | |
|  | **HR** | ***P*** | **95% CI** | **HR** | ***P*** | **95%CI** | **HR** | ***P*** | **95% CI** |
| HALP score (≤24.92 *vs*.>24.92) | 2.523 | 0.001* | 1.430-4.451 | 1.926 | 0.016* | 1.131-3.279 | 1.885 | 0.020* | 1.103-3.221 |
| Age (> 60 years *vs*. ≤60 years) | 1.350 | 0.311 | 0.750-2.427 | - | - | - | - | - | - |
| Gender (male *vs*. female) | 1.391 | 0.247 | 0.796-2.433 | - | - | - | - | - | - |
| Ann Arbor stage (stage III–IV *vs*. stage I–II) | 1.930 | 0.037* | 1.039-3.584 | 0.637 | 0.195 | 0.321-1.261 | 0.578 | 0.165 | 0.267-1.252 |
| ECOG physical status score (≥2 points *vs*. 0–1 point) | 2.212 | 0.010* | 1.206 -4.049 | - | - | - | - | - | - |
| LDH levels (> 250 U/L *vs*.≤250 U/L) | 2.941 | P<0.001* | 1.658-5.208 | 1.986 | 0.015* | 1.143-3.451 | 1.852 | 0.045* | 1.015-3.378 |
| Number of extranodal involvement (≥2 *vs*. 0–1) | 1.965 | 0.027* | 1.080-3.571 | 1.416 | 0.310 | 0.723-2.774 | 1.174 | 0.730 | 0.472-2.918 |
| IPI score (3–5 points *vs*. 0–2 points) | 3.268 | <0.001* | 1.842-5.814 | - | - | - | 1.430 | 0.554 | 0.437-4.674 |
| Hans classification (GCB subtype *vs*. non-GCB subtype) | 0.877 | 0.654 | 0.495-1.555 | - | - | - | - | - | - |

**Supplementary Table 2.** Analysis of prognostic factors for PFS in DLBCL patients

| **Indicator** | **Univariate analysis** | | | **Multivariate analysis** | | | **Multivariate analysis** | | |
| --- | --- | --- | --- | --- | --- | --- | --- | --- | --- |
|  |  | | | **withouts IPI score** | | | **plus IPI score** | | |
|  | **HR** | ***P*** | **95% CI** | **HR** | ***P*** | **95% CI** | **HR** | ***P*** | **95% CI** |
| HALP score (≤24.92 *vs*.>24.92) | 1.974 | 0.007* | 1.196-3.170 | 1.674 | 0.044* | 1.013-2.766 | 1.818 | 0.028* | 1.066-3.102 |
| Age (> 60 years *vs*. ≤60 years) | 1.599 | 0.077 | 0.951-2.689 | - | - | - | - | - | - |
| Gender (male *vs*. female) | 0.808 | 0.392 | 0.497-1.315 | - | - | - | - | - | - |
| Ann Arbor stage (stage III–IV *vs*. stage I–II) | 1.266 | 0.356 | 0.767-2.090 | - | - | - | - | - | - |
| ECOG physical status score (≥2 points *vs*. 0–1 point) | 1.585 | 0.110 | 0.901-2.790 | - | - | - | - | - | - |
| LDH levels (> 250 U/L *vs*.≤250 U/L) | 2.345 | P<0.001* | 1..435-3.834 | 2.113 | 0.004* | 1.276-3.500 | 1.880 | 0.039* | 1.031-3.429 |
| Number of extranodal involvement (≥2 *vs*. 0–1) | 1.645 | 0.069 | 0.962-2.812 | - | - | - | - | - | - |
| IPI score (3–5 points *vs*. 0–2 points) | 2.083 | 0.003* | 1.285-3.389 | - | - | - | 1.143 | 0.703 | 0.575-2.270 |
| Hans classification (GCB subtype *vs*. non-GCB subtype) | 1.019 | 0.942 | 0.615-1.687 | - | - | - | - | - | - |

**Supplementary Table 3.** Predictive performance of prognostic models for different survival endpoints

| **Endpoint** | **Model** | **1‑year‑dependent**  **AUC** | **3‑year‑dependent**  **AUC** | **3‑year Brierscore** |
| --- | --- | --- | --- | --- |
| OS | HALP+LDH | 0.4259 | 0.7475 | 0.199 |
|  | IPI | 0.5463 | 0.6669 | 0.206 |
|  | HALP+IPI | 0.5000 | 0.7345 | 0.194 |
| PFS | HALP+LDH | 0.6560 | 0.6870 | 0.256 |
|  | IPI | 0.5914 | 0.6024 | 0.269 |
|  | HALP+IPI | 0.6148 | 0.6498 | 0.264 |

**Supplementary Table 4.** Mean and median values of survival time

| **Variables** | **B** | ***SE*** | **Wald** | **DF** | ***P*** | **Exp(B)** | **95% CI for Exp(B)Lower** | **95% CI for Exp(B)Upper** |
| --- | --- | --- | --- | --- | --- | --- | --- | --- |
| HALP-LDH model risk | 1.184 | 0.367 | 10.388 | 1 | 0.001 | 3.268 | 1.591 | 6.716 |
| Ann Arbor stage | 0.214 | 0.421 | 0.257 | 1 | 0.612 | 0.808 | 0.354 | 1.843 |
| Number of invaded lymph nodes | 0.643 | 0.370 | 3.025 | 1 | 0.082 | 1.903 | 0.922 | 3.928 |
